# Pleural effusion as an atypical presentation of Kawasaki disease: a case report and review of the literature

**DOI:** 10.1186/s13256-019-2284-4

**Published:** 2019-11-25

**Authors:** Elif Arslanoglu Aydin, Selcan Demir, Orkun Aydin, Yelda Bilginer, Seza Ozen

**Affiliations:** 1 Department of Pediatrics, Hacettepe University Faculty of Medicine, 06100 Ankara, Turkey; 2 Department of Pediatric Rheumatology, Hacettepe University Faculty of Medicine, 06100 Ankara, Turkey; 3 Department of Pediatric Emergency, Hacettepe University Faculty of Medicine, 06100 Ankara, Turkey

**Keywords:** Kawasaki disease, Pleural effusion, Pulmonary involvement

## Abstract

**Background:**

Kawasaki disease is an acute, febrile vasculitis of childhood that affects medium-sized arteries, predominantly the coronary arteries. It is a multisystem disease; therefore, it may present with non-cardiac findings of disease.

**Case presentation:**

Here, we report the case of 7-year-old Turkish girl who presented with symptoms of fever, chest pain, and vomiting, who was diagnosed as having Kawasaki disease. We also present a literature review on pulmonary involvement due to Kawasaki disease.

**Conclusion:**

Pediatricians should consider the diagnosis of Kawasaki disease in the presence of pneumonia and pleural effusion that is nonresponsive to antibiotic therapy. This will prevent delay in diagnosis and the adverse consequences of the disease.

## Background

Kawasaki disease (KD) is one of the most common vasculitis disorders of childhood [1]. Although it is a multisystem disease that mainly affects the coronary arteries, it can, rarely, present with unusual system involvement of the pulmonary system, gastrointestinal tract, central nervous system, and genitourinary system [1]. Here, we report the case of a patient with KD who presented with an unusual form of pleural effusion. We also present a literature review on the subject.

## Case presentation

**Fig. 1:**
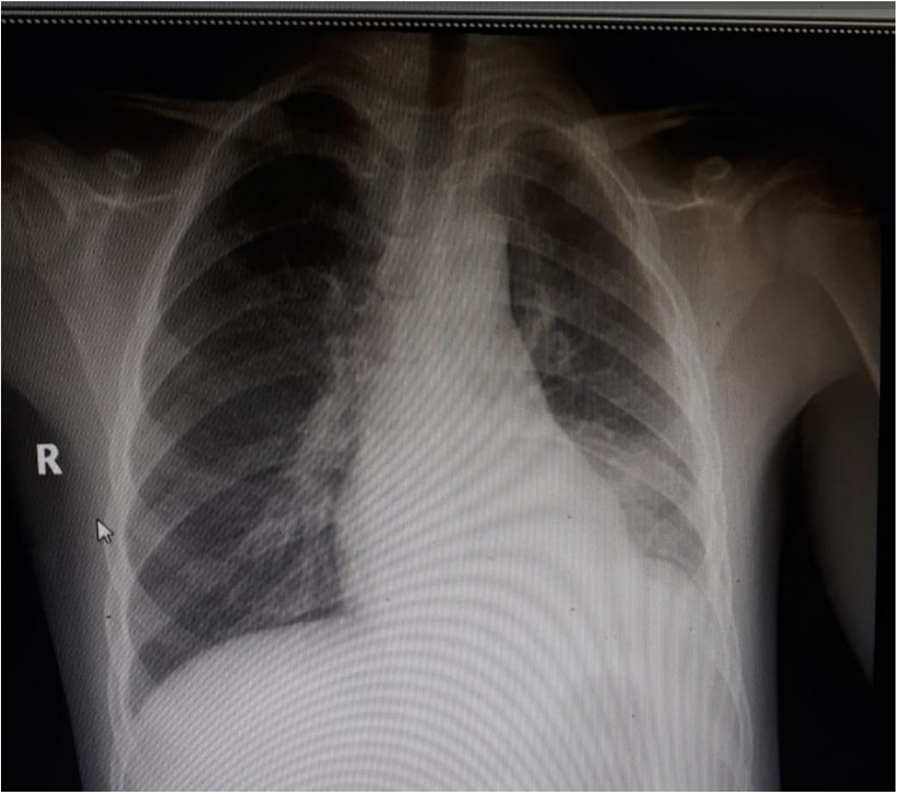
Chest X-ray of the patient showing left lower lobar consolidation with pleural effusion

A 7-year-old Turkish girl presented to a local hospital with fever, chest pain, and vomiting. At hospital admission, she was febrile with a respiratory rate of 50 per minute. On physical examination, auscultation of her lungs revealed diminished breath sounds of the lower lobe of her left lung. An anteroposterior (AP) chest X-ray and chest ultrasonography showed a left lower lobar consolidation with minimal pleural effusion. She was hospitalized and sulbactam ampicillin (SAM), ceftriaxone, and clarithromycin were initiated. On the third day, her condition worsened with increasing pleural effusion (Fig. 1). Thoracentesis was performed. SAM and ceftriaxone treatments were discontinued and meropenem and vancomycin were started. A chest tube was inserted and 130 mL of pus was drained. Light’s criteria were positive for an exudative pleural effusion; a pleural fluid culture was sterile. After 4 days, the chest tube was removed. High fever persisted for 15 days despite broad spectrum antibiotics, and acute-phase reactants remained high; therefore, she was referred to our hospital for further evaluation.

She had a fever with a temperature of 38.1 °C, her respiratory rate was 48/minute, heart rate was 125/minute, blood pressure was 90/65 mm Hg, and oxygen saturation was 95%. A physical examination revealed non-purulent conjunctivitis in both eyes, perianal peeling, and periungual desquamation on her hand, fingers, and toes. All other findings in the physical examination were unremarkable. She had unilateral cervical lymphadenopathy and a rash on her extremities while in the other hospital. Her past medical history was unremarkable, as was her family history. Immunizations were up-to-date for her age.

On admission to our hospital, the laboratory findings were as follows: hemoglobin 10.2 g/dL, white blood cells 14,000/μL, and platelets 736,000/μL. C-reactive protein (CRP) was 4.26 mg/dL (normal, 0–0.8 mg/dL), the erythrocyte sedimentation rate (ESR) was 42 mm/hour (normal, 0–20 mm/hour), and the albumin, creatinine, aspartate aminotransferase, alanine aminotransferase, gamma glutamyl transferase, blood urea nitrogen, calcium, sodium, chloride, and potassium levels were normal. Urine analysis was normal.

A chest X-ray was normal. Perivascular brightness and echogenicity of her right coronary artery was noted on transthoracic echocardiography (TTE). She was diagnosed as having KD based on the presence of fever, bilateral non-purulent conjunctivitis, cervical adenopathy, perianal peeling, periungual desquamation, elevated acute-phase reactants (ESR, CRP), thrombocytosis, and coronary artery involvement (CAI). Intravenous immunoglobulin (IVIG) (2 g/kg, infusion in 12 hours) and acetylsalicylic acid (60 mg/kg per day) were initiated. The fever resolved after IVIG infusion. At a 3-month follow-up visit, the acute-phase reactants and a TTE were normal. One year after the diagnosis, a TTE was normal and she was perfectly healthy.

## Discussion and conclusion

The most important complication of KD is CAI, which leads to enlargement, aneurysm, ischemic heart disease, and sudden death [1]. The clinical course of KD is highly variable. There are no pathognomonic clinical or laboratory findings to help diagnose KD. The diagnosis of KD in this case was made using the criteria of the American Heart Association [1]. In the presence of at least 5 days of fever, if there are at least four of the five principal criteria (cervical adenopathy, bilateral non-purulent conjunctivitis, oropharyngeal mucosal changes, polymorphous rash, erythema of the palms or soles, and edema of the hands or feet) the patient is diagnosed as having KD [1].

KD may present with uncommon symptoms such as pneumonia, pleural effusion, diarrhea, vomiting, sterile pyuria, gallbladder hydrops, acute cholestatic hepatitis, arthritis, and aseptic meningitis [2–7]. Pulmonary system involvement of KD is very rare; KD can present as pneumonia, pulmonary nodules, bronchopneumonia, hydropneumothorax, and pleural effusion [6, 8, 9]. Singh *et al.* showed that 1.3% of patients had pulmonary involvement and pleural effusion was seen in 54.5% of these patients [6]. Ugi *et al*. reported the case of an adult patient who presented with pulmonary involvement, specifically bilateral massive pleural effusions [10]. Occasionally, pleural effusion may be associated with bacterial agents such as *Mycoplasma pneumoniae* and *Streptococcus* [11, 12]. Pulmonary symptoms are mostly initially treated with antibiotics. However, if fever and accompanying signs ensue, the diagnosis of KD should be considered. Patients with pulmonary involvement may be more likely to have CAI due to delays in diagnosing KD and administration of IVIG [12–17].

We performed a review of the literature using PubMed and the search terms: Kawasaki disease AND pulmonary involvement; OR Kawasaki disease AND pulmonary presentation; OR Kawasaki disease AND pleural effusion. The searches were limited to the English language and pediatric patients. Case series and single case reports involving pediatric patients with KD with pulmonary involvement were included. Inconsistencies were resolved through discussion with the author SO, who also reviewed the literature. The authors EAA and OA searched the literature and manually screened titles and abstracts for relevance. Inconsistencies were resolved through discussion with the author SO.

**Fig. 2:**
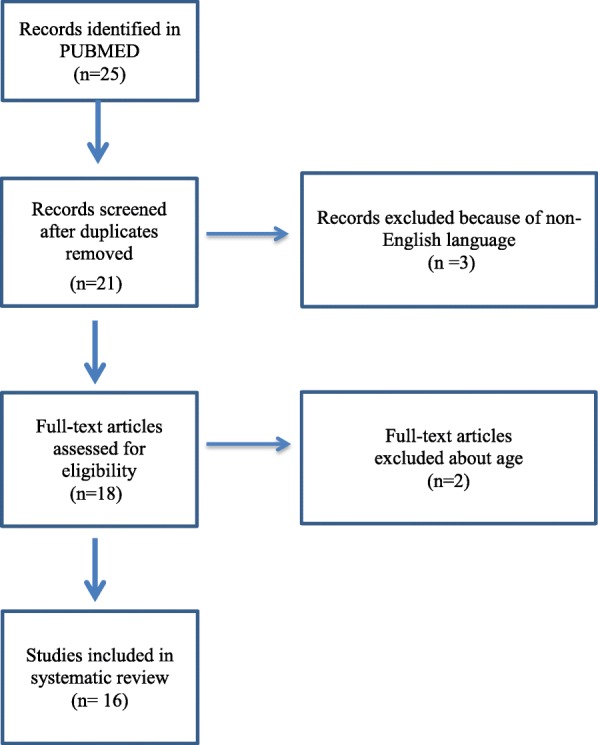
Study flowchart

Figure 2 lists the schematic analyses of the systematic literature review. At first, 25 related articles were found, but nine articles were excluded because of duplication, non-English language, and adult age, which left 16 articles [6, 8, 11–24]. The characteristics of these patients are summarized in Tables 1, 2, and 3. Finally, 20 patients with pleural effusions due to KD were identified [6, 11–18, 20, 24]. Of the 20 reviewed patients, TTE results were available in nine patients and seven had CAI [6, 12–17, 24]. Eleven patients presented with respiratory symptoms such as cough, dyspnea, and tachypnea [6, 12–15, 20]. Only four patients [14, 17, 18, 24] had complete KD, 10 patients [6, 13, 15, 16, 20] had incomplete KD, and six patients’ [11, 12] presentations were not available. Although a definite infectious agent could be shown for two patients [18, 24], all of the patients received antibiotics except one [14]. Two patients [6, 17] received a second dose of IVIG, and five patients received a second dose of IVIG and corticosteroid treatment for KD [13–16, 18].

**Table 1:** Clinical symptoms and laboratory parameters of patients who had pulmonary involvement associated with Kawasaki disease

Authors, year, reference number	Rash	Oral changes	Extremity changes	Red eyes	Adenitis	Other clinical symptoms	Hb (g/dL)	WBC (/mm^3^)	Plt (/mm^3^)	CRP (mg/dL)	ESR (mm/hour)
Singh *et al*., 2018, [6]	5^*a*^	1 ^*a*^	8^*a*^	1 ^*a*^	0 ^*a*^	Perianal desquamation, 3 ^*a*^; irritability, 1 ^*a*^	NA	25,009^*b*^	886,545^*b*^	14.05^*c*^	53.75 ^*d*^
Alhammadi and Hendaus, 2013, [12]	NA	NA	NA	NA	NA	NA	NA	24,000	600,000	10	65
Lee *et al*., 2011, [11]	NA	NA	NA	NA	NA	NA	NA	NA	NA	NA	NA
Lee *et al*., 2010, [14]	Yes	Yes	Yes	Yes	Yes	Perianal desquamation	NA	5500	178,000	2.8	21
Falcini *et al*., 2009, [15]	No	No	No	Yes	No	Irritability	9.5	21,800	710,000	27.8	99
Elizabeth *et al*., 2007, [16]	No	Yes	Yes	No	Yes	Irritability	10.2	12,800	550,000	7.7	86
Yavuz *et al*., 2007, [17]	Yes	Yes	Yes	Yes	No	Non-pigmented keratic precipitates in both of the patient’s eyes, sterile pyuria	10.4	32,800	734,000	21.8	90
Sittiwangkul and Pongprot, 2004, [13]	Yes	No	Yes	Yes	No	Irritability	NA	21,200	231,000	4.77	66
de Magalhães *et al*., 2012, [21]	Yes	Yes	Yes	Yes	No	Induration at the BCG site, perianal desquamation	6.5	25,000	905,000	34	120
Hamada *et al*., 2005, [18]	Yes	Yes	Yes	No	Yes	Hepatomegaly	NA	17,800	NA	13.9	NA
D'Souza *et al*., 2006, [20]	No	No	Yes	No	Yes	Sterile pyuria	9.8	56,800	690,000	NA	138
de Maddi *et al*., 2009, [22]											
Case 1	No	Yes	No	Yes	Yes	No	8.7	11,200	561,000	10.4	70
Case 2	No	No	No	No	No	No	9	26,960	142,000	40.8	107
Case 3	Yes	Yes	No	Yes	No	Irritability, sterile pyuria	11	18,500	1,087,000	2.95	50
Freeman *et al*., 2003, [23]											
Case 1	Yes	Yes	No	Yes	No	Irritability	NA	NA	1,120,000	NA	NA
Case 2	No	Yes	No	Yes	Yes	Torticollis	NA	NA	1,102,000	NA	114
Case 3	Yes	No	No	No	Yes	Anorexia	NA	NA	450,000	10.5	NA
Kobayashi *et al*., 2006, [24]											
Case 1	Yes	Yes	No	Yes	Yes		9.6	13,000	321,000	12.6	102
Case 2	Yes	Yes	Yes	Yes	No	Induration at the BCG site	11.6	18,800	314,000	7.6	NA
Vaidya *et al*., 2017, [8]	Yes	Yes	Yes	No	No		8.9	15,600	567,000	5.6	40
Akagi *et al*., 2017, [19]											
Case 1	Yes	Yes	Yes	No	No		NA	NA	NA	4.26	NA
Case 2	Yes	Yes	No	No	No		NA	NA	NA	4.32	NA

^a^Number of the patients who had the symptom

^b,c,d^Values are expressed as mean for 11, 8, and 10 patients, respectively

*BCG* Bacille Calmette–Guérin, *CRP* C-reactive protein, *ESR* erythrocyte sedimentation rate, *Hb* hemoglobin, *NA* not available, *Plt* platelet, *WBC* white blood cell

**Table 2:** Demographic parameters and clinical presentations of patients who had pulmonary involvement associated with Kawasaki disease

Authors, year, reference number	Patients (*n*)	Sex	Age at onset of disease (months)	Initial symptoms	Fever duration (days)	Chest X-ray findings
Singh *et al*., 2018, [6]	11	F, 6 ^*a*^; M, 5 ^*a*^	30 ^*b*^	Fever, cough, tachypnea	14.1 ^*b*^	Consolidation,11 ^*a*^; pleural effusion,6 ^*a*^; empyema, 3 ^*a*^; pneumothorax, 2 ^*a*^
Alhammadi and Hendaus, 2013, [12]	1	F	36	Fever, cough, sore throat	18	Consolidation, pleural effusion
Lee *et al*., 2011, [11]	54	NA	NA	NA	NA	Reticulonodular,17 ^*a*^; opacification, 34 ^*a*^; consolidation, 12 ^*a*^; pleural effusion, 5 ^*a*^ diffuse interstitial, 5 ^*a*^; atelectasis, 2 ^*a*^;
Lee *et al*., 2010, [14]	1	M	22	Fever, cough, rhinorrhea	6	Infiltration, pleural effusion
Falcini *et al*., 2009, [15]	1	F	30	Fever, cough,	12	Pleural effusion
Elizabeth *et al*., 2007, [16]	1	F	36	Fever, gum bleeding	21	Pleural effusion
Yavuz *et al*., 2007, [17]	1	M	11	Fever, pharyngeal erythema, dyspnea	> 5	Pleural effusion
Sittiwangkul and Pongprot, 2004, [13]	1	F	11	Fever, jaundice, diarrhea, dyspnea	15	Pleural effusion
de Magalhães *et al*., 2012, [21]	1	F	3	Fever	10	Infiltration
Hamada *et al*., 2005, [18]	1	F	60	Fever, abdominal pain, knee joint pain	15	Pleural effusion
D'Souza *et al*., 2006, [20]	1	M	5	Fever, diarrhea, dyspnea	7	Pleural effusion
Case 1	1	M	8	Fever, sore throat	8	Consolidation
Case 2	1	F	11	Febrile seizure, sore throat, cough	14	Consolidation
Case 3	1	F	23	Fever, cough	10	Consolidation
Freeman *et al*., 2003, [23]						
Case 1	1	M	4	Fever, cough, rash	21	NA
Case 2	1	M	6	Fever	60	Normal; thorax CT, pulmonary nodule
Case 3	1	NA	5	Fever cough, rash	4	Infiltration, multiple pulmonary nodules
Kobayashi *et al*., 2006, [24]						
Case 1	1	F	24	Fever, cracked lips, rash	5	Infiltration, pleural effusion
Case 2	1	F	24	Fever, cough, nasal discharge	4	Atelectasis
Vaidya *et al*., 2017, [8]	1	F	3	Fever, rash, dyspnea	32	Hydropneumothorax, consolidation, pneumatoceles
Akagi *et al*., 2017, [19]						
Case 1	1	F	4	Fever, erythema of the lips, rash	NA	NA; thorax MRI, bilateral multiple pulmonary nodules
Case 2	1	F	5	Fever, erythema of the lips, rash	9	Infiltration; thorax CT, bilateral pulmonary nodules

^a^Number of the patients who had noted findings

^b^Values are expressed as mean for 11 patients

*CT* computed tomography, *F* female, *M* male, *MRI* magnetic resonance imaging, *NA* not available

**Table 3:** Treatment, coronary artery involvement, follow-up, and outcomes of patients who had pulmonary involvement associated with Kawasaki disease

Authors, year, reference number	Infectious agent	Antibiotic treatment	CAI	Treatment	Follow-up and outcome
Singh *et al*., 2018, [6]	2 ^*a*^	11 ^*a*^	3 ^*a*^	2 ^*a*^, Second dose of IVIG	9 ^*a*^ Normal, 2 ^*a*^ NA
Alhammadi and Hendaus, 2013, [12]	No	Yes	Yes	IVIG	Normal
Lee *et al*., 2011, [11]	NA	NA	NA	NA	NA
Lee *et al*., 2010, [14]	No	No	Yes	Second dose of IVIG and corticosteroid	Normal
Falcini *et al*., 2009, [15]	No	Yes	Yes	Second dose of IVIG and corticosteroid	Normal
Elizabeth *et al*., 2007, [16]	No	Yes	Yes	Second dose of IVIG and corticosteroid	Normal
Yavuz *et al*., 2007, [17]	No	Yes	Yes	Second dose of IVIG	Normal
Sittiwangkul and Pongprot, 2004, [13]	No	Yes	Yes	Second dose of IVIG and corticosteroid	Aneurysm persisted in 2 years
de Magalhães *et al*., 2012, [21]	No	Yes	Yes	Second dose of IVIG, corticosteroid, MTX, and ETN	Aneurysm decrease but persisted
Hamada *et al*., 2005, [18]	No	Yes	No	Second dose of IVIG and corticosteroid	Normal
D'Souza *et al*., 2006, [20]	No	Yes	No	IVIG	Normal
de Maddi *et al*., 2009, [22]					
Case 1	No	Yes	No	IVIG	Normal
Case 2	No	Yes	No	Not given IVIG	Normal
Case 3	No	Yes	No	IVIG	Normal
Freeman *et al*., 2003, [23]					
Case 1	No	Yes	Yes	IVIG	Death
Case 2	NA	Yes	Yes	IVIG	Normal
Case 3	No	Yes	Yes	IVIG	Normal
Kobayashi *et al*., 2006, [24]					
Case 1	Yes	Yes	NA	IVIG	Normal
Case 2	Yes	Yes	NA	IVIG	Normal
Vaidya *et al*., 2017, [8]	No	Yes	Yes	IVIG	NA
Akagi *et al*., 2017, [19]					
Case 1	No	No	Yes	IVIG	Normal
Case 2	No	Yes	Yes	IVIG	Normal

^a^Number of the patients

*CAI* coronary artery involvement, *ETN* etanercept, *IVIG* intravenous immunoglobulin, *MTX* methotrexate, *NA* not available

In this case, our patient initially had an exudative, noninfectious pleural effusion and no response to antibiotics. CAI was also noticed and IVIG was administered on the 15th day of fever. After IVIG treatment, our patient’s clinical and laboratory findings improved dramatically, and the fever and acute-phase reactants returned to normal. It remains unclear as to whether the KD was triggered by the infection of the pleural space or if the pulmonary finding was a feature of the inflammation of KD.

KD can affect various systems as well as the coronary arteries, and may present with an unusual clinical picture. The diagnosis of KD with atypical presentations may be difficult for pediatricians. Early diagnosis and treatment can prevent complications.

## Data Availability

Data sharing is not applicable to this article because no datasets were generated or analyzed during the current study.

## Abbreviations

AP: Anteroposterior
CAI: Coronary artery involvement
CRP: C-reactive protein
ESR: Erythrocyte sedimentation rate
IVIG: Intravenous immunoglobulin
KD: Kawasaki disease
SAM: Sulbactam ampicillin
TTE: Transthoracic echocardiography
